# Threatened edible insects in Hidalgo, Mexico and some measures to preserve them

**DOI:** 10.1186/1746-4269-2-51

**Published:** 2006-12-04

**Authors:** Julieta Ramos-Elorduy

**Affiliations:** 1 Departamento de Zoología, Instituto de Biologia, Universidad Nacional Autónoma de México, México, DF

## Abstract

Edible insects are a natural renewable resource that provides food to many ethnic groups in Mexico. Some of these species are overexploited because of increased consumption, caused by the huge human population growth in the area and because of the large demand of these insects from many restaurants in Mexico and in other countries.

In Tulancalco, a small arid village in the State of Hidalgo, I carried out studies on edible insects over 25 years. The inhabitants of this village have a natural economy and use some 30 species of insects as food. At present, we have noticed a decrease in the population of several species due to overexploitation, which is carried by non-qualified independent workers who are not natives of the town. These gatherers sell their catch to make a living, thus contributing to the socioeconomic factors associated with this issue. These actions have degraded the ecosystems of this area, and consequently the prevention of these measures is critical.

The study species in this paper include 14 threatened species and we discuss some pragmatic measures that could implemented to avoid their extinction. In addition, some actions for the preservation of the ethnoentomobiodiversity in the area are proposed.

## Background

**Figure 1:**
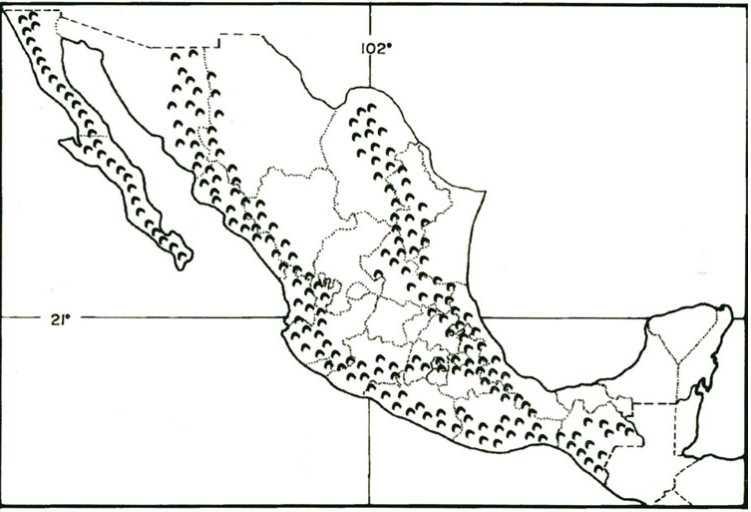
Orography of Mexican Republic.

A great part of the Mexican territory is covered by mountains which are home to a variety of habitats, ecological niches and communities [1] (Fig. 1), many of which have been completely isolated for a long time. People living in these areas have relied on gathering resources from their surroundings, and have maintained their ecosystem, not only for the source of food and medicine but also for the spiritual values that they conferred to the different resources [2-6]. As the access into these sites increased, inhabitants gained access to all kinds of different products, many of which are processed products; consequently, the lifestyle and diet of these people have been modified.

Posey [7] stated that 90% of the germ plasm of the planet was preserved by different ethnic groups. Traditions and beliefs have been kept in some aspects of their lives [8], but resources which urban inhabitants demand are often found in rural areas. The purchase of these resources provides an income that the natural or subsistence economy of the countryside cannot supply. Thus, this trade not only allows residents to consume processed products but advertisements for different products also makes them think that they are enjoying a better life style because they consume what they consider prestigious food. We suggest that this is a social type of pollution that has modified their feeding patterns.

Migration to the country's capital or abroad is a common phenomenon which must be considered in the present study [6]. Mainly young, male residents constitute the human capital of the community and abandon rural areas, leaving behind the elderly and children. This creates a new paradigm of concepts and values in the rural communities, which consequently drifts further away from tradition, and finally leads to a loss of traditional knowledge [6].

As rural workers grow older and try to satisfy their new psychological needs they abandon the fields in search of jobs in the city and forget their origins, their autochthonous food, and, consequently, their means of acquiring resources.

This lack of interest and jobs in rural areas allowed unqualified people to capitalize on the demand and lack of supply for edible insects for the market. Consequently, they exploit the resources in an irrational way, deteriorating the environment and depleting the resources. The situation is exacerbated by the desire for certain insect species from the middlemen or from monopolies of this industry.

Originally, indigenous people gathered edible insects for consumption, sharing it among their families, and only selling the surplus on certain occasions. Due to pressure and the hope of satisfying other needs (clothing, footwear, dishes, nails, etc.) they began overexploiting this resource [9] to fill the demand of these edible insect species both in Mexico and abroad[10].

Generally, indigenous people who migrate to Mexico City return on the weekends to spend time with their families. Since they know about the demand and the price paid for edible insects from being in the city, they quickly harvest large quantities of these resources without respecting the conditions required by these species to thrive. This, together with the demographic explosion of the country and the economic crisis in which it is submerged, has increased this immoderate exploitation.

Nowadays, insects are considered to be a non-domesticated resource since few species are cultivated. According to research, 40 species of edible insects in Mexico are presently in danger (Ramos-Elorduy, personal observation).

Ethnobiology has a great social responsibility, not only through the study of humans and their relationship with nature, but also through the responsibility of denouncing the deterioration that humans inflict on resources in order to prevent such damage in the future. Ethnobiological studies are multidisciplinary and inter-institutional since these studies link many disciplines, including Entomology, Ecology, Biology, Anthropology, Sociology, Economy, History, Geography and Ethnobiology. The latter was the first science to join man and the environment in a pragmatic way, mostly focusing on the preservation of species, and is based on the interests of the local population.

**Figure 2:**
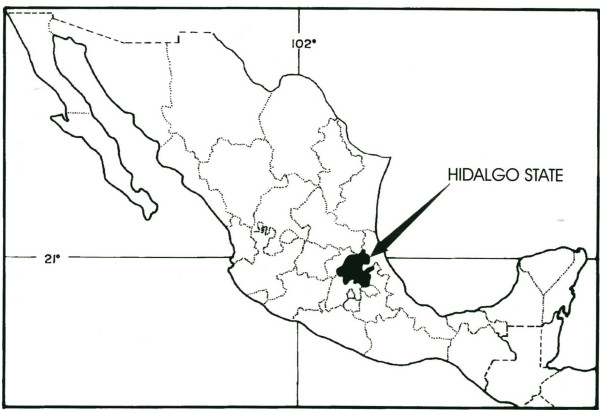
Geography situation of Hidalgo State in Mexican Republic.

Here, we will discuss the case of a town in the State of Hidalgo (Fig. 2), which has for some years engaged in entomophagic activity and the exploitation of insect resources. We will evaluate how the pressure of exploitation has finally created an "alert" in various species and the possibilities of avoiding further resource depletion.

### Study zone

Tulancalco, Hidalgo is a small town a few kilometers from Mexico City with 3,000 inhabitants of Nahuatl (2/3) and Otomi (1/3) origin. Located at the base of the Sierra Madre Oriental (20°20' north latitude and 99°20' east longitude) [11]. It is a mountainous area with both small and large valleys at an altitude of 2,400 meters and with a C (Wo) climate The region is characterized by a dry climate (the driest of the temperate subhumid classification), summer rains and a P/T quotient under 43.2 [12]. The subsistence economy provides low income for inhabitants; many do not earn more than about $8.00 USD a month. The nutritional status of the inhabitants is classified as bad and very bad, and symptoms of endemic malnutrition are found throughout over the state. Hunger and malnutrition prevail because of the very low caloric-protein intake [13].

The ecosystem is classified as Xerophytic bush [1] and is mainly composed of "mezquites" (*Prosopis juliflora *(Sw.) DC), "pirul" (*Schinus molle *L.), nopal (*Opuntia *spp.), maguey (*Agave *spp.), wild lettuce (*Agave lechuguilla *T.), "palo loco" (*Senecio praecox *(Cav.) D.C.), "jarilla" (*Senecio salignus *D.C.) and "garambullo" (*Myrtillocactus geometrizans *(M.). Besides shrubs like the "barredero" (*Baccharis conferta *HBK), there are many herbaceous plants of *Eupatorium*, *Vigueira *and *Zaluzania *genus and various gramineous during the rainy season.

We have frequently visited this town during many years. Its inhabitants were workers of an old Hacienda producing "pulque" (fermented drink of water-honey agave), which after the Mexican Revolution became their property. Initially, the land was divided among the families that worked there, but the land was continually divided further when children reached adulthood, and now each property is so small that it cannot provide enough to live on. This situation, together with the decreased popularity of Mexican "pulque", caused the replacement of agave plants with corn or other crops, and modifying their environment further.

## Methods

This study is based on 25 years of field work carried out in different states of the Mexican Republic. We went to Tulancalco every month for three years, to determine which insect species the inhabitants ate, and how they preserved these species. Later, we went back twice a year to evaluate the status of some species of edible insects. These stays consisted of visits to the nests of the recorded *Liometopum apiculatum *M. and *Myrmecosistus melliger *W. or hives of *Polybia occidentalis nigratella *B. to evaluate their condition. We also measured the number of organisms of white and red agave or "botija" worms (*Aegiale hesperiaris *W.*, Comadia redtembacheri *H.*, Scyphophorus acupunctatus *G.) and xamues (*Thasus gigas *B.) in an area of about 300 Km^2^, and observed the status of the aquatic bugs of the Corixidae family which produce the "Ahuauhtle" in water ponds or lagoons.

## Results

**Figure 3:**
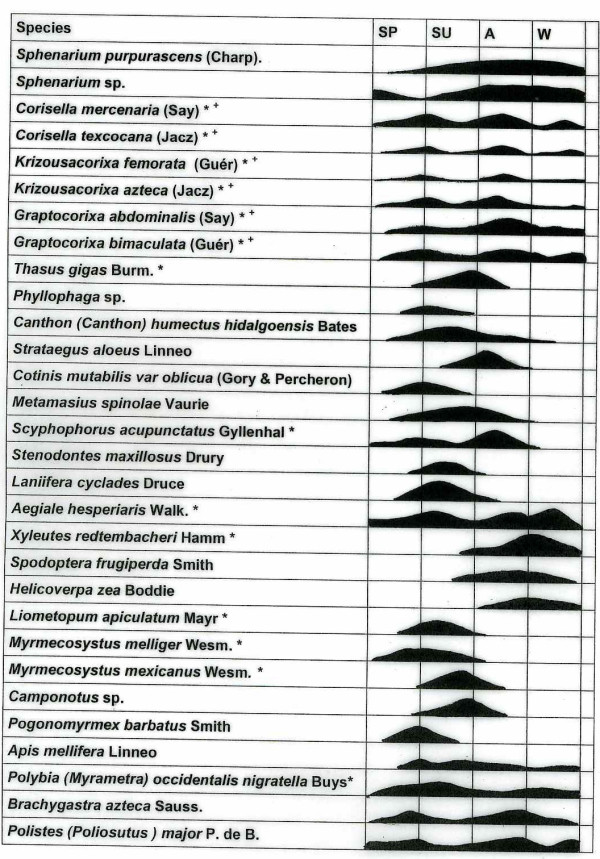
Seasons of presence and abundance of edible insects in Tulancalco, Hidalgo, Mexico.

In Tulancalco, five main orders of insects are consumed. Most of these species are terrestrial (79%) and only a few aquatic 21%; (Table 1), while, 48.39% are threatened (14 species) six threatened species are aquatic. Together, these species comprise 31.62% of the 102 species recorded in the state of Hidalgo [14]. Both univoltine and polivoltine species are consumed. It is possible to find the latter throughout the year, but univoltines vary depending on the season. Hence, the capture of these insects depends on their life cycle, and on their presence and abundance throughout the year (Fig. 3). Qualified gatherers used to gather these species only when they were most abundant, and this allowed for reproduction during the less abundant periods. However, present over-exploitation has caused these species to be in danger of extinction in this region. Among the most prominent of the threatened species, are the "escamoles", "gusanos blanco y rojo del agave", the "botija", the "xamues", the "ahuahutle" and "axayacatl", the "vinitos" and the avispa negra, which has been available as a resource for more than 500 years. [15,16].

**Table 1:** Edible insects consumed in Tulancalco Hidalgo, México.

**Order**	**Family**	**Species**
Orthoptera	Acrididae	*Sphenarium purpurascens *(Charp).
		*Sphenarium *sp.
Hemiptera	Corixidae	*Corisella mercenaria *(Say) * ^+^
		*Corisella texcocana *(Jacz) * ^+^
		*Krizousacorixa femorata *(Guér) * ^+^
		*Krizousacorixa azteca *(Jacz) * ^+^
		*Graptocorixa abdominalis *(Say) * ^+^
		*Graptocorixa bimaculata *(Guér) * ^+^
	Coreidae	*Thasus gigas *Burm. *
Coleoptera	Scarabaeideaea	*Phyllophaga *sp.
	Scarabaeidae	*Canthon (Canthon) humectus hidalguensis*
	Melolonthidae	*Strategus aloeus *Linneo
		*Cotinis mutabilis var oblicua*
	Curculionidae	*Metamasius spinolae *Vaurie
	Cerambycidae	*Scyphophorus acupunctatus *Gyllenhal *
		*Stenodontes maxillosus *Drury
Lepidoptera	Pyralidae	*Laniifera cyclades *Druce
	Megathymidae	*Aegiale hesperiaris *Walk. *
	Cossidae	*Xyleutes redtembacheri *Hamm *
	Noctuidae	*Spodoptera frugiperda *Smith
		*Helicoverpa zea Boddie*
Hymenoptera	Formicidae	*Liometopum apiculatum *Mayr *
		*Myrmecosystus melliger *Wesm. *
		Myrmecosystus mexicanus Wesm. *
		*Camponotus *sp.
		*Pogonomyrmex barbatus *Smith
	Apidae	*Apis mellifera *Linneo
	Vespidae	*Polybia (Myrametra) occidentalis nigratella *Buyson*
		*Brachygastra azteca *Sauss.
		*Polistes (Poliosutus) major *P. de B.

* Threaten species. + Aquatic species

There is a huge gap between the knowledge of the gatherers and that of the scientists, because many of gatherers do not know how to maintain these resources at a sustainable level.

### Causes of population decrease. "Escamoles" (*Liometopum apiculatum *M.) (Hymenoptera-Formicidae)

The "escamoles" (Dolichoderinae ants of the genus *Liometopum*) were part of the tributes for the Aztec Emperor Moctezuma [16], and their exploitation has persisted throughout time. Presently, its abundance in the area has decreased substantially. A long time ago, this resource was exploited in specific sites in Mexico during certain periods of the year because of its abundance and popularity. At this time, nests were exploited only by the "escamoleros", groups of trained men who knew where to find them and, after opening the nests and retrieving the insects, were careful enough not to harm the nest so that the ants could continue to be exploited during future seasons [17].

However, due to their delicious taste, and the market demand, the current price for these insects is high ($200.00 USD/K). Gatherers with no knowledge of the species look for and collect these insects in large numbers for sale. They look for new nests in many sites and exploit them carelessly. This has decreased the productivity of the "escamoles", because the ants must work much more. Some nests have disappeared due to extreme modification of conditions, a particular misfortune because a nest could continue to produce and be lucrative for 40 years even when exploited up to three times during the harvesting season [18]. Assuming that every larvae or pupae in its reproductive stage weighs around 0.0384 grams and a worker cast weighs 0.025 g., we can calculate the number of individuals extracted from the number of tons that are sold.

Nowadays, not only "real" escamoles are exploited (the immature stage of the reproductive breed), but also the working cast. This leaves the colony without enough workers to preserve the nest. Without the critical biomass, these nests will not produce more "escamoles" until this balance is reestablished or the nest dies.

Recently at San Juan market in Mexico City, monopolizers (2004) informed us that small airplanes loaded with tons of the product arrived from the United States and sold it to the highest bidders. This is no surprise because the species is also found in the states of Colorado, New Mexico and Texas. It is unclear whether these insects are exploited in the U.S. by Mexicans living there or by Americans.

### White agave worm (*Aegiale hesperiaris *Walk.) (Lepidoptera-Megathymiidae)

**Figure 4:**
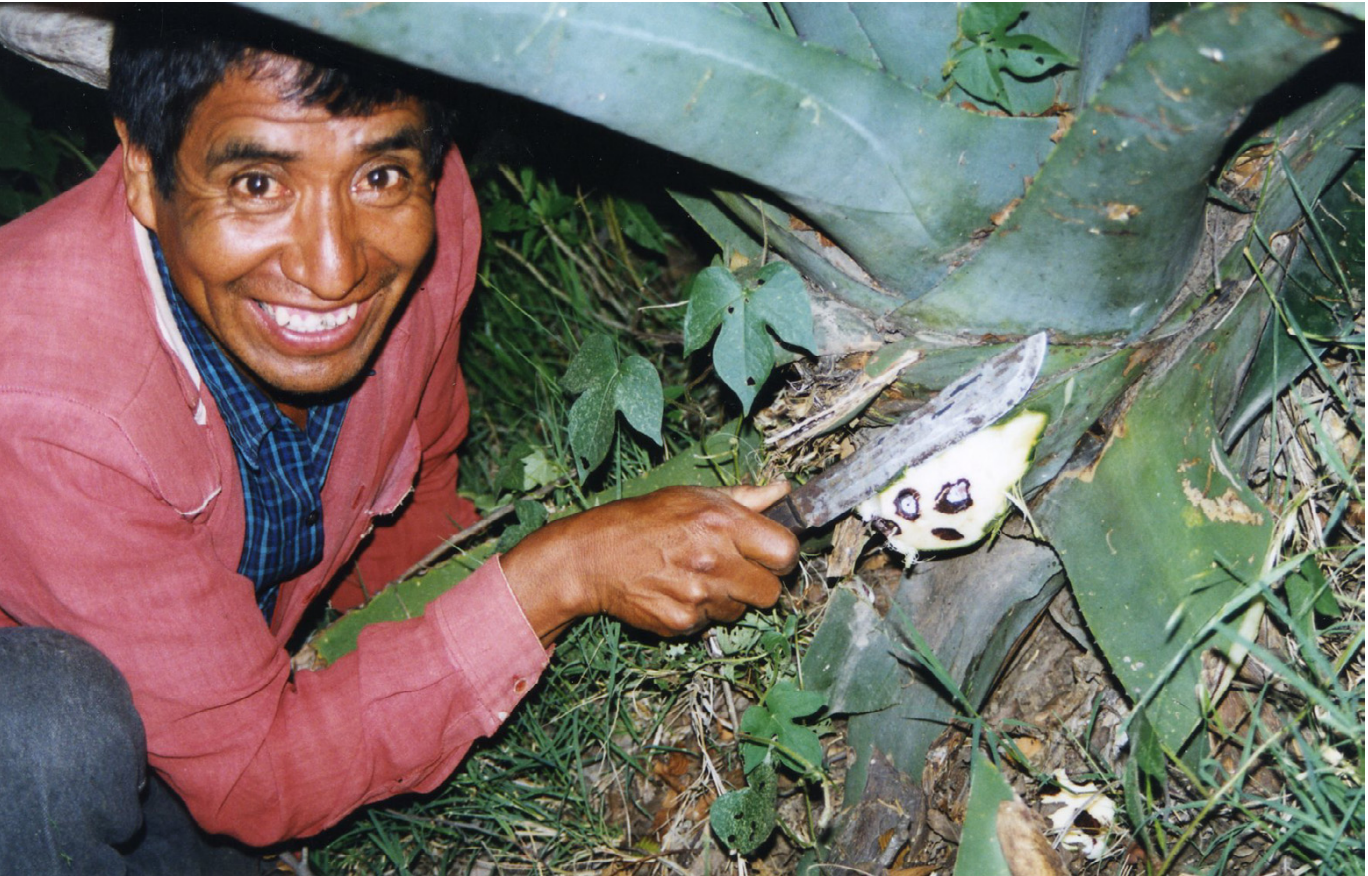
Peasant cutting fleshy leave of an agave plant.

The larval stage of this insect has a great demand in Mexico because it is a traditional dish and because of its refined and exquisite taste, peculiar and difficult to characterize. It shelters in the fleshy leaves of 1 m to 1.5 m large "pulque"-producing agaves *Agave atrovirens *K. ex S-D, this species is called "manso" and/or "cimarrón". There is either one larvae per leave and/or few larvae are found in different leaves of the same agave (Fig. 4). Due to the significant decrease of this species, agaves remain only as a natural fence around maize or barley crops. These crops are continuously sprayed with insecticides, and those substances reaching agaves kill most of the larvae.

In addition, a polyembryonic wasp (*Telonomus *sp.) which is an egg parasite, has decreased the worms in great numbers in some locations. Diverse predators like woodpeckers (*Melanoterpes *sp.) which drill into wood and then arrive sow bugs (Crustacea, Isopoda), to eat the larvae. Afterwards a mealybug (*Pseudococcus *sp.) that found favorable conditions to develops inside the gallery, penetrate together with different species of ants (*Formica *sp., *Camponotus *sp., *Myrmecosistus *spp.) that feed on the secretions of this mealybug, favoring also the growth of mushrooms and bacteria that causes diseases.

So, in addition to anthropogenic depredation, this species is also attacked by different predators. Hence, nowadays these larvae are found in smaller quantities and in sites that are farther from rural activity, and consequently gatherers have to travel greater distances to obtain only few specimens [19,3].

Due to its life cycle, 3 generations overlap and small, medium and large larvae are found concurrently. The latter stage provides the greatest profit but is found in the smallest amount. Gatherers do not understand that because of their harvesting techniques, the insects cannot reach an adult stage and reproduce.

With such a high demand in the market (sold at $250 USD/Kg.) and with such a low supply, gatherers have gradually substituted these insects with other species. The larvae of the *Opuntia *worm (*Laniifera cyclades *D.), which live in the wild or are found in *Opuntia *plantations cultivated for their fruits in dry zones, are sometimes substituted for *Aegiale hesperiaris*. The corn worm (*Helicoverpa zea *B.) and the ant soldier worm (*Spodoptera frugiperda *S.) are also gathered for this purpose. However, someone familiar with the species can easily detect the fraud, but restaurant owners or household members often do not notice it.

In one marketing study, done with 50 restaurants, in Mexico City, there was deficit of 7 1/2 tons of white agave worm [20].

### Red agave worm (*Xyleutes redtembacheri *Hamm.) (Lepidoptera-Cossidae)

Most of the young of the red agave worm are also found in the agave that produces pulque, but they are also found in several other species (e.g. *A. atrovirens *K. ex S-D, *A salmiana *O ex S-D, *A mapisaga *T.). Because of the lifestyle of the red agave worm and because of the way in which it is gathered, the agave is killed during the harvest of this species. This is because the worms live together (40 to 60 larvae) in the center of the stem, called "mezontete", located at the union with the fleshy leaf, so all of the stem and leaves must be removed during the gathering of these worms, causing the death of the agave. The larvae found are in the same stage and are about the same size, since they come from the same oviposition.

These larvae are also used for a Mexican alcoholic traditional drink called "mezcal" which has quite a large demand, even at an international level. The container holds a large worm, and if the worm is not included people do not buy it. These larvae are also part of the "worm salt" which is taken with the "mezcal". This is why the "mezcal" companies monopolize them, and they demand a large number of worms. The domestic market demand for these worms has also increased, thus decreasing their numbers. The kilo is sold for $200.00 USD in the market.

### "Botija" or "chatita" (*Scyphophorus acupunctatus *Gyllenhal). (Coleoptera-Curculionidae)

The weevil *Scyphophorus acupunctatus *is also found in the agave "mezontete", but only in those that have already been scraped for "agua miel" (water-honey), which, once fermented, produces "pulque". To be scraped, the agave has to be harvested just before flowering. It takes approximately 12 years for the plants to reach this stage, and the plants then die shortly after flowering. The number of agaves currently exploited to gather *S. acupuncatutus *is significantly lower than for *Aegiale hesperiaris*. This species is not a commercial item (it is consumed or exchanged for other resources), and it provides an important amount of tryptophan, the amino acid which is lacking in the rural diet of Mexico, and which has a high chemical qualification [21].

### Xamues (*Thasus gigas *Burm.) (Hemiptera-Coreidae)

*Thasus gigas *live in the "mezquite" tree (*Prosopis juliflora *(S) DC), their only host, and are also known as the "mezquite worm". The species was very abundant in this area, but now that a larger number of adults and larvae are gathered for sale rather than for personal consumption, the abundance of this species has declined. This species is univoltine, and it is collected in the spring [14]. Elderly people in the study area who eat this species, say that they can currently only find few specimens at a time, allowing a one to two month supply, while in the past they could gather enough to feed on for six months. They refer to this species as the "meat we eat". This insect is greatly appreciated because of its sweet taste, which is rare in the rural areas. After they are gathered, the insects are dried on a griddle, stored in plastic mesh bags, and hung in the kitchen where they provide a daily meal.

### Ahuahutle and axayacatl (*Corisella mercenaria *(Say), *C. texcocana *(Jacz), *Krizousacorixa femorata *(Guér), *K. azteca *(Jacz), *Graptocorixa abdominalis *(Say), *G. bimaculata *(Guér)). (Hemiptera-Corixidae)

The group of aquatic insects collectively called "Axayacatl" has been consumed and cultivated back to the prehispanic era. Their eggs are called "ahuahutle", and the cultivation of these species is very simple and costs little money because it can be undertaken with local practices [22]. During the holy week traditions, these species sell for the highest price. The Axayacatl are in danger, because of the actual bodies of water in the area themselves have dried up and because of pollution caused by garbage and other waste materials [23]. In addition, improper cultivation techniques by the inhabitants contributes to the decline of these species.

### "Vinitos" or "repletas" (*Myrmecosistus melliger *W., *M. mexicanus *W.) (Hymenoptera-Formicidae)

*Myrmecosistus melliger *and *M. mexicanus *are ants that are found in flat areas of arid zones, and their nests are built underground. They feed on plant or animals secretions (flowers, yolks and mealybugs) and are more abundant during the rainy season, which is when they are harvested. Through trophallaxis, *M. melliger *and *M. mexicanus *pass their food onto ants known as "repletas", which hang within a cavity in the center of the nest, at a depth of about 80 cms [24]. The "repletas" store secretions via abdomen distention so that the colony has food when it is scarce and pass the food on in the same way that the workers feed the young, the queen and themselves.

These ants are eaten alive, mostly by children who enjoy their sweet flavor. Once the "repletas" are extracted, the colony is left without any reserves on which to survive, and often the colony dies. These ants are only harvested for consumption, and they are normally consumed "in situ".

### Black wasp (*Polybia (Myrametra) occidentalis nigratella*) Buysson. (Hymenoptera-Vespidae)

The wasp *Polybia *(*M) occ. nigratella *is extensively distributed throughout the Mexican Republic, but is becoming scarce in the study area, because insect gatherers consume the whole hive, regardless of the size, which does not allow reproduction. The species is not only used as food; their venom is also used to cure rheumatism, arthritis and hysteria, and the honey also has a medicinal use for ophtalmic, cataracts and "pterigiones" [4].

Entire hives are sold in the markets all over Mexico once they are big enough.

Population trends of this species from 1980 to 2006 are summarized in Table 2.

**Table 2:** Quantity variation obtained (1980–2006) of threaten edible insects in Tulancalco, Hidalgo in an area of 100 Km^2^

Species	Number	1980	1990	2000	2006
*L. apiculatum *M.	Nests	8	7	4	2
*A. hesperiaris *W.	Organisms	39	112	85	43
*C. redtembacheri *H.	Organisms	480	283	247	180
*S. acupunctatus *G	Organisms	120	132	84	52
*Th. gigas B*.	Organisms	7000	5315	4200	3008
*M. melliger W*.	Organisms	280	178	87	30
*P. (M.) occidentalis nigratella *B.	Hives	25	17	12	8
*Axayacatl *(Corixidae bugs)	weight [Kg]	1.5	1.3	0.424	0.210

These results are the mean of two replicates each time.

## Discussion

Because edible insects are not considered by the national government as a "really" important food resource, they are not included in the laws for environmental regulation or included in international agreements on Biodiversity Conservation. Currently, these species are only considered to be susceptible of collection and/or sale for institutional or amateur scientific collections and some species can be sold for exorbitant prices for jewelry, arts and crafts, etc. Concerns regarding their harvest for use as food is not taken into account and should be established. We suggest that the following actions should be taken:

a. The development of "Edible Insects Exploitation and Trading Norms and Legislation" to avoid extensive depredation of these organisms and to remove the gap between the knowledge of the resources and their exploitation [25];

b. The development of "reserve zones" to be protected and to prohibit collections within these zones in order to preserve the germ plasm of the alimentary ethnoentomobiodiversity, and to prevent the erosion of the genetic pool of these insects;

c. The formation of a co-operative society as a means of mercantile production to regulate exploitation and avoid participation of middlemen;

d. Organization of academic courses, with the participation of Biology Departments of several diverse institutions and the schools in the area, at a town, state or capital level, and with the cooperation of specialists of different resources through educational courses (Entomology, Ecosystems, Ecology, Sampling Methods, etc.), information courses (about the resources), and training courses (about sampling, hosts, population dynamic, parasites, recognizing the status of the species, etc.);

e. Creation of a trust with donations from altruistic Institutions, whose subsidy has been authorized by the state government;

f. Synthesis of information on the most outstanding resources in terms of exploitation, and proper means of handling, protecting, and preserving insect species. Specifically, this would describe how to cultivate the most desired species and would implement prototypes;

g. Diffusion of information with objective talks on local radio stations, and through comic style flyers with allusive drawings, printed in local languages;

h. Promoting the importance of implementing maintenance cultivation techniques to scholars from multiple institutions within their area of expertise and for the most desirable species;

i. Increasing the insects "protocultivation" discussed in the paper. For omnivorous "escamoles", the number of individuals could be increased, by increasing food resources along their paths or close to their nests to preserve the species. The number of nests per unit area could also be increased, establishing new natural foundations [26], or carrying out the culture placed on their spot [27]. After exploitation, proper care should be provided to the nests by replacing part of the nest opening (the trabeculae) [17], adding dry *Opuntia *or *Agave *fleshy leaves, covering it with a big handful of fresh weed, large stones, mud and finally camouflaging it.

The technique we implemented with the white agave worm in a 10 m^2 ^area we obtained 199 larvae [28]. According to our experiences in working with the red agave worm, after removing the stem with the lips of the agave and collecting the red worm, immediately restoring the same plant allows 79% of agave plants to survive. It is necessary to reestablish parcels to grow agave plants. To preserve the axayacatl, the prehispanic technique culture must be taught to all the inhabitants living closer to water ponds ("*jaqueyes*") and lagoons. To preserve the black wasp, simple shelters with wooden planks could be built and placed as a two-sided roof. These could be placed in homes and neighboring areas to let the wasps grow until they mature [29]; and

j. Formal controlled introduction of these species into the markets with public advertisements and open exposure.

These steps require effort, but could be implemented progressively.

Evans [30] stated that "the commercialization of one species, is the way to achieve its preservation" since obtaining an economic gratification causes the harvesters to take an interest in preserving the resource for the acquired benefit.

People living in Tulancalco and in most towns in this and other States of the country, can be classified as poor farmers with an income that rarely exceeds $1,000.00 per year ($90.00 USD). These farmers work under a monetary deficit, with high auto consumption rates, and who make their living as employers or from small business [31]. This farming economy takes place in small scale production units where production relationships without a salary predominate. Thus, there are no possibilities for accumulating capital and their purpose is not to maximize a profit but to guarantee their subsistence [32].

These people who are agricultural day-laborers, proletariats without enough land, get poorer and poorer and lack all the nutritional support that the edible insects can provide. The capitalist sector grows richer by the marketing these edible species through middlemen who sell products to the industry and/or to restaurant owners.

The Center of Agricultural Investigations of Mexico has demonstrated that 84% of all the Agricultural property of the country is classified as common public land. This land ownership is classified as infra-sustainable or sub-familiar and it is small in size, and does not generate jobs or sufficient income to satisfy the minimum needs of a farmers' family. Farmers therefore have to look for jobs to survive since the production units are based on family work with no salary.

Additionally, the ecosystems in which these peasants live, (xerophytic bushes), are generally mountainous arid forest areas and/or unproductive mountainous areas. For this reason, edible insects and other resources of the area constitute products of crucial importance in their life, so the survival and abundance of these resources are essential to the harvesters' lives.

Apiculture is part of the farming activity of this area, and it has become a highly productive industry. Exporting bee products has allowed the country to obtain more than 35 millions of dollars per year, according to the Agriculture Ministry of México [33]. In this case, not only the honey is used, but the brood and in some populations the adults are also consumed. This is a model of what an agro-industry of other edible insect species could be. This type of farming should not only be taken as an alternative for economic wellbeing for the land and cattle land sectors, but also as a nutritional alternative at the service of the people. Edible insects can provide a source of high nutritional value, which have components that are missing in the daily diet of the people inhabiting these areas and which are highly digestible [3,6].

The development of these cultures should keep the technical and social aspects of the issue in mind; without a fair social policy, harvesting of these resources could increase the current crisis in the fields.

The capitalist marketing of soy-beans produced such an increase in the price of this resource, and consequently, the local people needing protein did not have the economic capacity for their consumption. This meant that many people could not even to afford to buy enough food to ensure the minimum calories or proteins required for their diet. Adequate food for the poor would be possible by rationally taking advantage of the natural resources provided by edible insects. Protein provided by edible insects would not only decrease social problems, but would also help Mexicans to have a more active participation in the economy and in national policies.

## Conclusion

Insects constitute an endless source of protein [34-36] due to their number and abundance. Many insects do not compete with man for their resources, and so do not affect the productivity of human or animal food and, with the exception of one species studied (red agave worm) never alter the production of their hosts (this species only affects its host because of the means of exploitation). Edible insects are highly nutritious because they store a considerable amount of protein and energy [36,37], are easily digested [38], and also have a pleasant taste.

Cultivation of these insects can be considered to be what Schumacher [39] denominates as intermediate technology, which requires only the resources on site.
